# Elevated P-Element-Induced Wimpy-Testis-Like Protein 1 Expression Predicts Unfavorable Prognosis for Patients with Various Cancers

**DOI:** 10.1155/2021/9982192

**Published:** 2021-12-31

**Authors:** Kehua Jiang, Tao Ye, Juan Du, Lanlan Tang, Xiaolong Chen, Fa Sun, Xifeng Sun

**Affiliations:** ^1^Department of Urology, Guizhou Provincial People's Hospital, Guiyang 550002, China; ^2^Medical College of Guizhou University, Guiyang, China; ^3^Integrated Medical Services of Tongji Hospital of Tongji Medical College of Huazhong University of Science and Technology, Wuhan 430030, China; ^4^Department of Urology, Medical University of Graz, Graz 8036, Austria

## Abstract

Increasing evidence has shown that overexpression of P-element-induced wimpy-testis (PIWI)-like protein 1 (PIWIL1) was associated with unfavorable prognosis of patients with various types of cancers. Herein, we conducted this meta-analysis to identify the clinicopathological and prognostic value of the PIWIL1 expression in cancers. Three electronic databases (PubMed, Web of Science, and Embase) were comprehensively retrieved for relevant studies up to August 4^th^, 2019. RevMan 5.3 and STATA 12.0 statistical software programs were used to explore the relationships between PIWIL1 expression and the prognosis and clinicopathological features in cancer patients. A total of 13 studies recruiting 2179 patients with 9 types of solid tumors were finally included in the meta-analysis. The results indicated that patients with high PIWIL1 expression tended to have a shorter survival, and additionally deeper tumor invasion, higher clinical stage, and more lymph node metastasis. PIWIL1 could serve as a biomarker for prognosis and clinicopathological characteristics in various cancers.

## 1. Introduction

Nowadays, cancer is a major public health problem worldwide and has become one of the leading causes of death and the biggest obstacle to improving average lifetime. With rapid population growth and aging worldwide, the incidence and mortality of cancers have greatly increased [1, 2]. Numerous researches have studied the mechanisms of the occurrence and development of various cancers, and great progress has been achieved in the prevention, diagnosis, and treatment. However, the five-year overall survival rate is still relatively low in the majority of cancer patients [1]. Therefore, studying the specific mechanisms behind tumorigenesis and tumor development has become more popular and significant.

Initially, the PIWI gene was found as P-element-induced wimpy testis mutation which hindered germline stem cell division in *Drosophila melanogaster* in 1970 [3]. The PIWI proteins, a subfamily of argonaute proteins, have been detected in various species [4, 5]. PIWIL1, also called HIWI, is one of the four human homologues of the PIWI family, which is cytogenetically mapped to 12q24.33 [6, 7]. It has been reported that the PIWI family are evolutionarily conserved and important in a series of biological processes, such as self-renewal and division of stem cell, spermiogenesis, RNA silencing, transposon silencing, and posttranscriptional regulation in several different organisms [6, 8–12].

The first report of PIWI expression in tumor tissue was in seminomas, in which Qiao et al. [7] found that PIWIL1 expression was positive in the tumor tissues but negative in the normal tissues; in addition, aberrant PIWIL1 expression might contribute to the occurrence and development of seminoma. PIWIL1 is the most studied protein among the PIWI family, which could regulate gene expression functioning in DNA damage response, cell cycle reentry, apoptosis, cell proliferation, and tight junctions [13–16].

Furthermore, the expression level of PIWIL1 has been found to be positively related to cell proliferation in several cancer cell lines [17]. Moreover, silencing PIWIL1 by siRNA could inhibit the expression of BCL2 and cyclin D1 and suppress cell proliferation by facilitating apoptosis in glioma cells [18]. Afterwards, emerging clinical evidences indicated that overexpression of PIWIL1 could be detected in different tumors including breast, colon, oesophageal, gastric, pancreatic, and hepatocellular carcinoma, and the expression of PIWIL1 was correlated with histological grade of tumor, clinical stage, and poorer clinical outcome of patients [19–22]. Positivity for PIWIL1 predicted chemoresistance in cervical cancer patients [23]. In pancreatic cancer, PIWIL1 facilitated metastasis via reducing cell-cell adhesion [24]. PIWIL1 maintains self-renewal and survival of glioma stem cells by regulating expression of related genes [25].

It is greatly urgent to determine novel molecular markers about cancers, which can contribute to more accurate risk stratification for cancer patients and better predicting tumor progression and the prognosis, as well as the prediction of the outcome of therapy and the development of personalized treatment based on the biological knowledge. Numerous studies have identified the overexpression of PIWIL1 gene/protein in various cancer types, suggesting that PIWIL1 might be involved in tumorigenesis or tumor progress [26, 27]. Given that the PIWIL1 is mostly expressed in the testis and broadly elevated in different cancers, PIWIL1 has the potential to be an ideal target for cancer diagnosis and therapy [7]. However, the universal adaptability of PIWIL1 to predict prognosis for cancers is still unclear. Therefore, a meta-analysis and systematic review was conducted to synthetically confirm the association between PIWIL1 expression and prognosis in various cancers.

## 2. Materials and Methods

### 2.1. Literature Search

Three electronic databases (PubMed, Web of Science, and Embase) were comprehensively retrieved up to August 4^th^, 2019. The combination of the following keywords was used in the literature search: (“PIWIL1” or “HIWI” or “Piwi-Like Protein 1” or “Piwi Like RNA-Mediated Gene Silencing 1”) and (“cancer” or “tumor” or “carcinoma”). In addition, the reference lists were also manually reviewed to obtain potential articles.

### 2.2. Inclusion Criteria

The included articles must meet the following inclusion criteria: (1) investigation based on human cancer; (2) studies reporting the associations of PIWIL1 expression with clinical outcomes (overall survival (OS), cancer-specific survival (CSS), disease-free survival (DFS), and recurrence-free survival (RFS)) and clinicopathological characteristics; (3) studies directly providing hazard ratios (HRs) with corresponding 95% confidence intervals (CIs) for survival information, or survival curves to extract these data using the method described by Tierney et al. [28]; (4) cancer patients divided into “high/positive” group or “low/negative” group. The following studies were excluded: (1) reviews, letters, or comments; (2) animal or cell experiment studies; (3) studies without sufficient information.

### 2.3. Data Extraction and Quality Assessment

Two authors carefully reviewed the full-text and independently extracted data following a previously designed form. Controversy was settled via discussion with a third author. The following information was extracted: first author, country, cancer type, number of patients analyzed, specimen type, method of measurement, cutoff value, HR estimated method and HR for survival (OS, CSS, or DFS/RFS), and clinicopathological characteristics (such as age, gender, tumor size, differentiation, tumor invasion, clinical stage, lymph node metastasis (LNM), and distant metastasis (DM)).

Besides, the quality of included studies was determined by Newcastle-Ottawa Scale (NOS) containing a total of 9 scores [29]. Study with more than 6 score was considered high quality.

### 2.4. Statistical Analysis

The prognostic value of PIWIL1 overexpression in cancer patients was appraised by combined HRs and corresponding 95% CIs. Combined HRs for OS, CSS, and DFS/RFS were calculated separately. The relationship between PIWIL1 overexpression and clinicopathological characteristics was assessed by pooled estimates of odds ratios (ORs) and 95% CIs. The statistical analyses were conducted using the RevMan5.3 and STATA 12.0 statistical software programs. Heterogeneity across publications was evaluated by *Q* test and I-squared test. *P* value<0.1 and *I*^2^>50% indicated significant heterogeneity, and a random-effect model would be used; otherwise, a fixed-effect model was preferred for the analysis. Begg's linear regression test was conducted to identify the potential heterogeneity factors. Sensitivity analysis was also conducted to check the stability of our results. *P* values less than 0.05 indicated statistical significance except for heterogeneity analysis.

## 3. Results

### 3.1. Search Results

**Figure 1:**
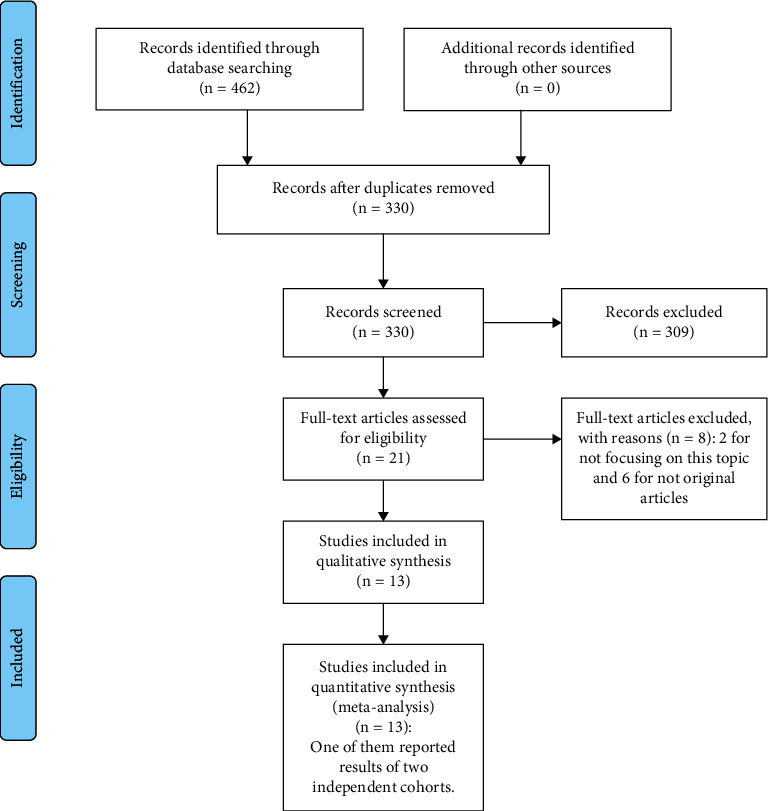
Flow diagram of literature research and selection process.

After systematic retrieval of the three previously mentioned databases, a total of 462 records were identified. The selecting process is detailed in Figure 1. Next, 132 duplicate articles were excluded, and 330 records remained for further assessment. After screening the title and abstracts, 309 irrelevant articles were eliminated, and the 21 potential studies were further checked by screening full texts. Finally, 13 studies were qualified for our meta-analysis [19, 21, 22, 30–39]. Significantly, study of Stöhr et al. [36] reported results of two independent cohorts of renal cell carcinoma patients, and in the subsequent analysis process, the two results were regarded as two studies.

### 3.2. Characteristics of Included Studies

A total of 2179 patients with 9 types of solid tumors including colorectal cancer (CRC), breast cancer, glioma, esophageal squamous cell carcinoma (ESCC), hepatocellular carcinoma (HCC), bladder cancer, renal cell carcinoma (RCC), gastric cancer (GC), and non-small cell lung cancer (NSCLC) from 13 eligible studies with concerned clinical outcomes were finally included in our meta-analysis. These articles were published between 2009 and 2019. Eight of the thirteen studies were conducted in China, two in Germany, one in Iran, one in Poland, and one in Spain. The number of the sample size ranged from 46 to 345, and seven studies enrolled more than 150 participants. All studies detected the PIWIL1 expression levels in tissue samples, and based on the expression levels, which were mainly detected by immunohistochemistry (IHC), these cancer patients were categorized into high/positive and low/negative expression groups in the included studies. Newcastle-Ottawa Scale (NOS) was used to evaluate the quality of these studies, and all eligible studies scored highly (>6). Twelve studies provided survival information, and eight studies reported clinicopathological characteristics. The main characteristics are summarized in Table 1.

### 3.3. Association between PIWIL1 Overexpression and Prognosis

**Figure 2:**
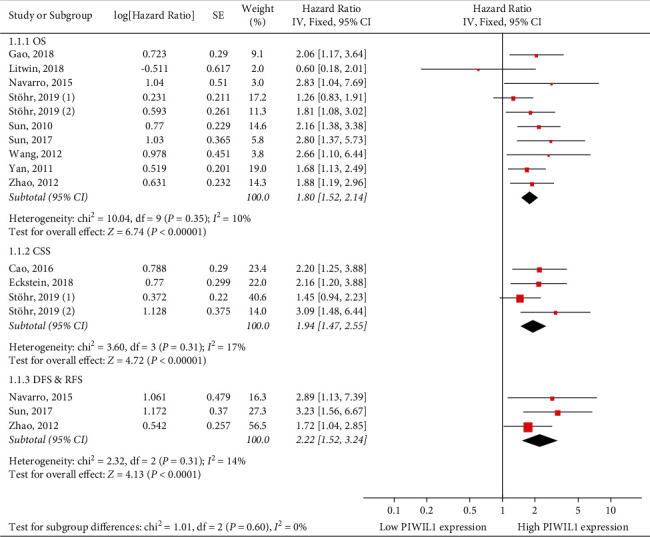
Forest plots of the association of PIWIL1 expression with OS, CSS, and DFS/RFS in various cancers.

Among the twelve studies evaluating the prognostic value of PIWIL1 overexpression in solid tumors, ten focused on OS, four on CSS, and three on DFS/RFS. As shown in Figure 2, a fixed-effect model was used to evaluate the pooled HRs with their 95% CIs due to no observation of significant heterogeneity. The pooled HRs were 1.80 (95% CIs: 1.52–2.14, *p* < 0.00001) for OS, indicating that PIWIL1 overexpression was significantly correlated with the reduced OS periods. Next, a meta-analysis for CSS was conducted, and the result revealed that higher PIWIL1 expression group was subjected to a shorter CSS outcome (HR = 1.94, 95% CIs: 1.47–2.55, *p* < 0.00001). We finally conducted a subgroup analysis based on OS, CSS, and DFS/RFS; the pooled HRs were 2.22 (95% CIs: 1.52–3.24, *p* < 0.00001). These results demonstrated that elevated PIWIL1 expression could predict unfavorable prognosis for patients with various cancers.

Additionally, we performed subgroup analyses for OS according to region, sample size, HRs extract method, and cancer type. Ultimately, similar results were obtained as regard to the effects of PIWIL1 overexpression on OS (Table 2).

### 3.4. Association of PIWIL1 Overexpression with Clinicopathological characteristics

**Figure 3:**
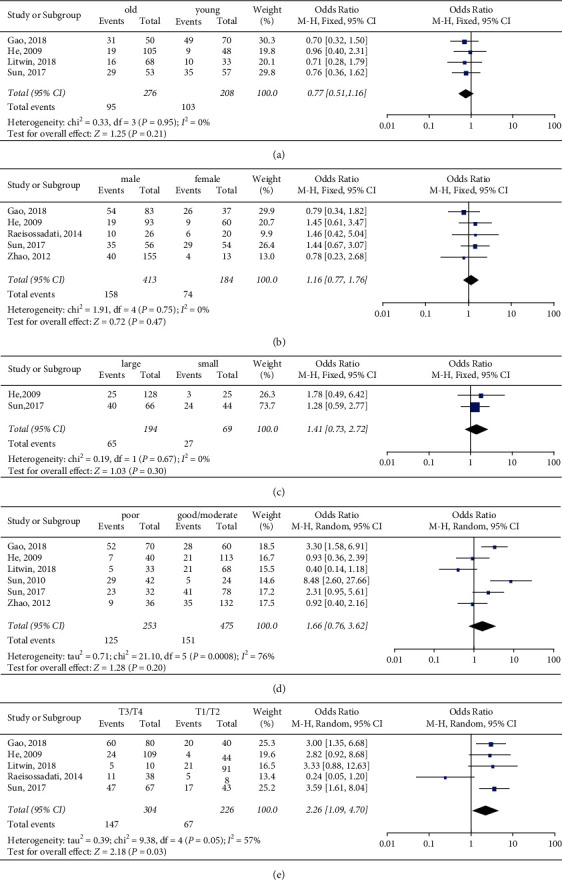
Forest plots of the association of PIWIL1 expression with(a) age, (b) gender, (c) tumor size, (d) differentiation, and (e) tumor invasion.

**Figure 4:**
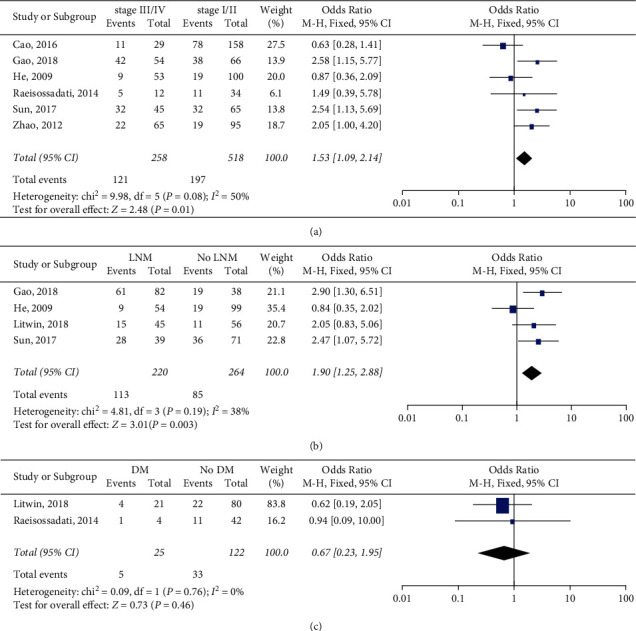
Forest plots of the association of PIWIL1 expression with (a) clinical stage, (b) lymph node metastasis (LNM), and (c) distant metastasis (DM).

Eight studies with 951 cancer patients were analyzed for the association of PIWIL1 overexpression with various clinicopathological characteristics; the pooled ORs are shown in Figures 3 and 4. The results suggested that PIWIL1 positive expression had no obvious relationship with age (*n* = 4, OR = 0.77, 95% CI: 0.51–1.16, *p*=0.21), gender (*n* = 5, OR = 1.16, 95% CI: 0.77–1.76, *p*=0.47), tumor size (*n* = 2, OR = 1.41, 95% CI: 0.73–2.72, *p*=0.30), differentiation (*n* = 6, OR = 1.66, 95% CI: 0.76–3.62, *p*=0.20), and distant metastasis (*n* = 2, OR = 0.67, 95% CI: 0.23–1.95). However, PIWIL1 positive expression was significantly associated with deeper tumor invasion (*n* = 5, OR = 2.26, 95% CI: 1.09–4.70, *p*=0.03), higher clinical stage (*n* = 6, OR = 1.53, 95% CI: 1.09–2.14, *p*=0.01), and more lymph node metastasis (*n* = 4, OR = 1.90, 95% CI: 1.25–2.88, *p*=0.003).

### 3.5. Analyses of Sensitivity and Publication Bias for PIWIL1 Expression and OS

**Figure 5:**
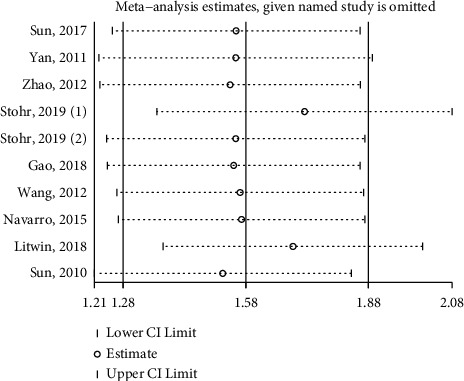
Sensitivity analysis plot of the association of PIWIL1 expression with OS.

**Figure 6:**
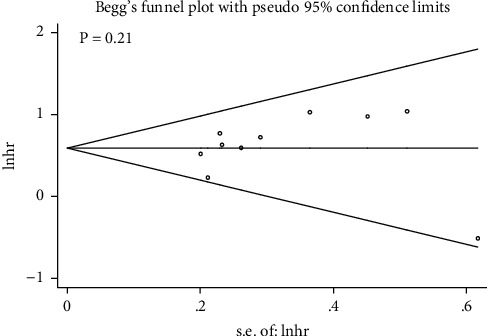
Begg's funnel plots of the association of PIWIL1 expression with OS.

Sensitivity analysis was used to evaluate the outcome stability of PIWIL1 expression and OS, and the result indicated that the pooled HRs were stable and credible (Figure 5). Begg's funnel plot was used to evaluate the publication bias, and no significant publication bias for OS was identified (*p*=0.21) (Figure 6).

## 4. Discussion

Overexpression of PIWIL1 had been discovered to facilitate cancer progression and predict poor prognosis of patients with various cancers. Plenty of clinical researches have explored the value of PIWIL1 overexpression to predict prognosis. However, almost all these researches, which included limited number of subjects of specific cancer, have come to incomprehensive conclusions.

This current meta-analysis is the first comprehensive review of all published clinical research in regard of the influence of PIWIL1 expression level on prognosis of 9 types of solid tumors. Survival data of 2179 cancer patients included in 13 different studies were systematically estimated. In summary, the overall results specifically suggested that high PIWIL1 expression was associated with poor prognosis in cancers, with results of poor OS (pooled HR = 1.80, 95% CIs: 1.52–2.14, *p* < 0.00001), poor CSS (pooled HR = 1.94, 95% CIs: 1.47–2.55, *p* < 0.00001), and poor DFS/RFS (pooled HR = 2.22, 95% CIs: 1.52–3.24, *p* < 0.00001). Additionally, subgroup analyses, according to region, sample size, HRs extract method, and cancer type, suggested that the relationship between high PIWIL1 expression and poor OS was significant. As for clinicopathological characteristics, the results suggested that PIWIL1 overexpression had no obvious relationship with age, gender, tumor size, differentiation, and distant metastasis, but was significantly associated with deeper tumor invasion (*n* = 5, OR = 2.26, 95% CI: 1.09–4.70, *p*=0.03), higher clinical stage (*n* = 6, OR = 1.53, 95% CI: 1.09–2.14, *p*=0.01), and more lymph node metastasis (*n* = 4, OR = 1.90, 95% CI: 1.25–2.88, *p*=0.003).

For now, this study is the most full-scale meta-analysis and systematic review which scientifically revealed the possible prognostic role of PIWIL1 expression level in cancers. The results convincingly confirmed the present main viewpoint that overexpression of PIWIL1 was associated with the OS, CSS, DFS/RFS, tumor invasion, clinical stage, and lymph node metastasis. What is more, two important implications were put forward in this study. Firstly, PIWIL1 overexpression could be a common poor prognostic biomarker in cancers. In this study, we involved 9 types of cancers, including breast cancer, CRC, glioma, ESCC, HCC, RCC, bladder cancer, GC, and NSCLC, which meant that the results were universal and this finding could be applied to at least these 9 types of solid tumors. Secondly, it signified the potential to exploit PIWIL1 as a worthy treatment target for solid tumors.

A lot of research has explored the action mechanisms of PIWIL1 on tumorigenesis and tumor progression in different cancers. It was widely confirmed that the PIWI proteins could bind to Piwi-interacting RNAs (piRNAs), which are 24–32 nt long, single stranded gonad-specific small interfering RNAs [40]. So, PIWIL1 could exhibit important roles in self-renewal and division of stem cell, gametogenesis, and regulating gene expression via RNA interfering mechanism [9, 12, 20]. piRNAs and PIWIL1 protein function as a Piwi-ribonucleoprotein complex to suppress transposon through target degradation and epigenetic silencing [41, 42]. In various cancer cells, high expressions of PIWIL1 and piRNAs lead to aberrant DNA methylation, tumor-suppressor genes silencing, and an abnormal “stem-like” state of cancer cells [43, 44]. Specifically, in human HCC, PIWIL1 expression was significantly higher in HCC tissue [45], and PIWIL1 played a critical role in HCC proliferation and metastasis by being mediated by small hairpin RNA [46]. Recently, Wang et al. reported the critical role of PIWIL1 in mediating the crosstalk of cancer cell metabolism and immune cell response of HCC, and they found that overexpression of PIWIL1 promoted the proliferation rate of human HCC; moreover, they revealed that PIWIL1 increased energy production and oxygen consumption through fatty acid metabolism without altering aerobic glycolysis, and PIWIL1 attracted myeloid-derived suppressor cells (MDSCs) into the tumor microenvironment (TME) and activated p38-MAPK signaling, which in turn improved secretion of immunosuppressive cytokine IL10 [47]. Similarly, the mature transcripts were associated with the PIWIL1-piRNA complex code critical regulatory proteins involved in controlling cell proliferation, differentiation, and survival in CRC cells, which actively contributes to the establishment and maintenance of clinicopathological characteristics of CRC [48]. The PIWIL1 expression in CRC is positively correlated with the mRNA level of OCT4, a cancer stem cell marker, suggesting that PIWIL1 may contribute to the tumor stemless, which in turn strongly improves its metastatic potential [49]. Although the underlying molecular basis of the oncogenic functions of PIWIL1 remains largely unknown, PIWIL1 has been recently reported to mediate the occurrence and progression of human cancers possibly through piRNA-independent mechanisms [24, 50]. Shi et al. revealed that PIWIL1 regulates mRNA expression through the UPF1-mediated nonsense-mediated mRNA decay pathway [51]. These findings have made the oncogenic mechanism mediated by PIWI proteins more comprehensive than the previously well-established PIWI-piRNA pathway.

In particular, CRC may develop in patients with distinct intestinal diseases such as inflammatory bowel diseases (IBD) and irritable bowel syndrome (IBS), suggesting that different TME can increase the risks of CRCs in different ways [52]. TME represents a complex network between tumor cells and endothelial, stromal, and immune cells [53]. Besides, the inflammatory cells and inflammatory mediators such as cytokines and chemokines in TME facilitate CRC progression [54]. Stimuli like inflammatory cytokines and growth factors play a critical part in cancer development by abnormally regulating the epithelial-mesenchymal transition (EMT) of cancer cells [55]. What is more, TME facilitates CRC progression by maintaining paracrine crosstalk signalings between tumor resident adipocytes [56]. Accumulating evidence has shown the critical role of intestinal barrier function regulated by mucus, IgA, and lipocalin2 in protecting from bacteria-induced inflammation and tumor tumorigenesis, and numerous signaling pathways (e.g., Toll-like receptors), metabolites (e.g., indole, bile acids), and small non-coding RNAs (e.g., miRNA, piRNA) have been identified as key regulators mediating interactions between host and microbe in the intestine [57]. In particular, there is growing evidence that the role of dysregulation of microRNAs (miRNAs) in the cancer development, progression, and metastasis is important with the silence effect, acting as tumor suppressors or oncogenes to posttranscriptionally regulate expressions of specific mRNA targets [58–60]. Recent evidence indicates that miRNAs are involved in direct cell-to-cell signaling and paracrine signaling between TME and tumor cells, acting as secreted molecules in microvesicles or exosomes [61]. Furthermore, several miRNA-target therapeutics have developed in clinical level, including a mimic of the tumor suppressor miR-34, which have reached phase I clinical trials for cancer treatment, providing a perspective on achieving safe miRNA therapy [62]. In CRC, there is mounting evidence indicating there are crosstalks between miRNAs and the Wnt/*β*-catenin signaling pathway [63], EGFR signaling pathways [64], TGF-*β* signaling pathway [65], TP53 signaling pathway [66], and the EMT [67] in progression and metastasis. For example, amplification of the AKT-PIK3K-PTEN signaling pathway is mediated by the downregulation of miR-1, miR-126, and miR-497 or by upregulation of miR-19, miR-19, and miR-96 [68]. In addition, research also has revealed the potential pathogenic mechanism of miRNAs via regulating apoptosis. For example, overexpression of miR-195 promoted apoptosis in colorectal cancer cell line via targeting antiapoptotic BCL-2 [69].

Interestingly, CRC screening has already a great impact on curbing the rising incidence of colorectal cancer [70]. CRC is the third most-commonly diagnosed cancer and the second in cancer mortality worldwide, and the incidence rates show wide geographical variations, with a 3-times higher rate in developed countries than in developing countries [2]. The pathogenesis of CRC follows a regular progression from benign adenomas to malignant adenocarcinomas and usually lasts more than 10 years. It is usually asymptomatic in the early stages and diagnosed until late stages with unfavourable prognosis and huge financial burdens [71]. The introduction of CRC population screening programs worldwide has significantly reduced CRC mortality in developed countries [72]. Colonoscopy is the current reference method for CRC screening and gold standard for CRC, and studies have shown that the sensitivity for detecting CRC is >95% [72] and a 53%–72% reduction in the CRC incidence and a 31% reduction in CRC-related mortality [73]. What is more, as for noninvasive CRC screening, faecal immunochemical test (FIT), with higher sensitivity and participation rate, is gradually replacing guaiac faecal occult blood test (G-FOBT) and has become the most-commonly used method for the global screening program. Multitarget stool DNA test (Cologuard) and plasma SEPT9 DNA methylation test (Epi proColon) are approved noninvasive tools but are not cost-effective with unsatisfactory accuracy [74]. In addition, the noninvasively detectable biomarkers such as proteins, DNA, miRNAs, low molecular weight metabolites, and CRC-related gut microbiome are being actively developed [75]. Offering various screening options, even with each patient's wishes and limitations, may increase compliance with screening [76]. These efforts to promote CRC screening have great potential to ultimately reduce CRC morbidity and mortality rate.

Accumulating evidence has shown the important role of regulatory T cells (Treg cells) in cancers [77, 78]. Immune cells in the premalignant environment can produce various cytokines, growth factors, chemokines, and proangiogenic factors, which contribute to a support environment by activating antiapoptotic pathways and neoangiogenesis and inhibiting immune surveillance [79]. Treg cells are a subset of T lymphocytes, which mediate the immune response by suppressing the proliferation and cytokine production of self-reactive T lymphocytes [80]. Evidence has indicated that Treg cells are recruited to TME through chemokines produced by cancer cells and, in particular, HCC cells have been found to secrete CCL5 and CCL28 chemokines to mediate accumulation of Treg cells [81]. Then, Treg cells regulate the activities of antigen presenting cells by expressing inhibitory costimulatory receptors on their surface to impair signaling between APCs and T cells [82]. Moreover, they also downregulate the expressions of CD40, CD80, and CD86 on dendritic cells and suppress the immune cells activity by secreting inhibitory cytokines such as IL-10, IL-35, and TGF-*β* [83]. Now, manipulation of Treg cells is a promising anticancer treatment strategy to facilitate Treg cell-targeted therapies and immune precision medicine [84].

However, our study has several limitations. Firstly, all included studies are retrospective and published with positive outcomes. Secondly, among the included studies, the methods assessing PIWIL1 expression and defining positive PIWIL1 expression are inconsistent. Thirdly, the sample size in the included studies is relatively small. Fourthly, data of a specific cancer is insufficient. Finally, the majority of subjects included in the study are from China, which may weaken the generalization of the conclusions.

## 5. Conclusions

To sum up, the association of high PIWIL1 expression in solid tumor tissues with poor survival was specifically certified in this meta-analysis. We suggested that high PIWIL1 expression level was a valuable predictor for poor cancer prognosis in deeper tumor invasion, higher clinical stage, and more lymph node metastasis. Therefore, PIWIL1 is a promising biomarker of worse clinical outcomes in cancers. But whether it would be a promising target for treating solid tumors still needs to be scientifically studied. Besides, further larger-scale and high qualified multicenter studies including different tumor types are required to confirm the clinical value of PIWIL1 expression in cancers.

## Figures and Tables

**Table 1:** Main characteristics of the included studies in this meta-analysis.

Study (year)	Country	Disease	Sample size	PIWIL1		Specimens	Method	Cutoff value	Outcome	Hazard ratios	Nos.
				High	Low						
Raeisossadati, 2014	Iran	CRC	46	16	30	Tissue	qRT-PCR	2 folds	CP	NR	7
Litwin, 2018	Poland	Breast cancer	101	26	75	Tissue	IHC	Score >8	OS, CP	SC	8
Sun, 2010	China	Glioma	66	34	32	Tissue	IHC	Score >3	OS, CP	SC	8
He, 2009	China	ESCC	153	28	125	Tissue	IHC	Score >4	CP	NR	8
Sun, 2017	China	CRC	110	64	46	Tissue	IHC	Intensity >1	OS, DFS, CP	MA	9
Cao, 2016	China	Breast cancer	187	89	98	Tissue	qRT-PCR	EI > 5	CSS, CP	SC	8
Yan, 2011	China	CRC	270	69	201	Tissue	IHC	>10%	OS	MA	9
Zhao, 2012	China	HCC	168	44	124	Tissue	IHC	Score >3	OS, RFS, CP	MA	9
Eckstein, 2018	Germany	Bladder cancer	95	37	58	Tissue	IHC	Score >2	CSS	MA	8
Stöhr, 2019 (1)	Germany	RCC	265	75	190	Tissue	IHC	Score >0	OS, CSS	MA	9
Stöhr, 2019 (2)	Germany	RCC	345	51	294	Tissue	IHC	Score >0	OS, CSS	MA	9
Gao, 2018	China	GC	120	80	40	Tissue	IHC	Positive cells >40%	OS, CP	MA	8
Wang, 2012	China	GC	182	NA	NA	Tissue	IHC	Score >3	OS	MA	7
Navarro, 2015	Spain	NSCLC	71	11	60	Tissue	qRT-PCR	Ct < 35	OS, RFS	MA	7

RC: colorectal cancer; ESCC: esophageal squamous cell carcinoma; HCC: hepatocellular carcinoma; RCC: renal cell carcinoma; GC: gastric cancer; NSCLC: non-small cell lung cancer; qRT-PCR: quantitative real-time polymerase chain reaction; IHC: immunohistochemistry; EI: expression index; Ct: cycle threshold; CP: clinical parameter; OS: overall survival; CSS: cancer-specific survival; DFS: disease-free survival; RFS: recurrence-free survival; NR: not reported; SC: survival curve; MA: multivariate analysis.

**Table 2:** Subgroup meta-analysis of pooled HRs for overall survival.

Variables	Studies (*n*)	Number of patients	HR (95% CIs)	*p* value	I2 (%)	Ph
(1) Overall survival	10	1698	1.80 (1.52–2.14)	<0.00001	10	0.35

(2) Region						
China	6	916	2.01 (1.63–2.48)	<0.00001	0	0.82
Europe	4	782	1.46 (1.09–1.97)	0.01	39	0.18

(3) Sample size						
≤150	5	468	2.12 (1.58–2.83)	<0.00001	22	0.28
>150	5	1230	1.66 (1.34–2.05)	<0.00001	0	0.53

(4) Extract method						
Survival curve	2	211	1.85 (1.21–2.82)	0.004	74	0.05
Multivariate analysis	8	1487	1.80 (1.49–2.17)	<0.00001	10	0.35

(5) Cancer type						
Gastrointestinal cancer	5	850	1.97 (1.55–2.49)	<0.00001	0	0.72
Other	5	848	1.97 (1.55–2.49)	<0.0001	42	0.14

HR: hazard ratios; CIs: confidence intervals.

## Data Availability

All data generated or analyzed in this study are included in this published article.
